# An innovative telemedicine knowledge translation program to improve quality of care in intensive care units: protocol for a cluster randomized pragmatic trial

**DOI:** 10.1186/1748-5908-4-5

**Published:** 2009-02-16

**Authors:** Damon C Scales, Katie Dainty, Brigette Hales, Ruxandra Pinto, Robert A Fowler, Neill KJ Adhikari, Merrick Zwarenstein

**Affiliations:** 1 Interdepartmental Division of Critical Care Medicine, University of Toronto, ON, Canada; 2 Department of Critical Care Medicine, Sunnybrook Health Sciences Centre, Toronto, ON, Canada; 3 Institute for Clinical Evaluative Sciences, Toronto, ON, Canada; 4 Center for Health Services Sciences, Sunnybrook Health Sciences Centre, Toronto, ON, Canada; 5 Department of Quality and Patient Safety, Sunnybrook Health Sciences Centre, Toronto, ON, Canada; 6 Department of Medicine, Sunnybrook Health Sciences Centre, Toronto, ON, Canada

## Abstract

**Background:**

There are challenges to timely adoption of, and ongoing adherence to, evidence-based practices known to improve patient care in the intensive care unit (ICU). Quality improvement initiatives using a collaborative network approach may increase the use of such practices. Our objective is to evaluate the effectiveness of a novel knowledge translation program for increasing the proportion of patients who appropriately receive the following six evidence-based care practices: venous thromboembolism prophylaxis; ventilator-associated pneumonia prevention; spontaneous breathing trials; catheter-related bloodstream infection prevention; decubitus ulcer prevention; and early enteral nutrition.

**Methods and design:**

We will conduct a pragmatic cluster randomized active control trial in 15 community ICUs and one academic ICU in Ontario, Canada. The intervention is a multifaceted videoconferenced educational and problem-solving forum to organize knowledge translation strategies, including comparative audit and feedback, educational sessions from content experts, and dissemination of algorithms. Fifteen individual ICUs (clusters) will be randomized to receive quality improvement interventions targeting one of the best practices during each of six study phases. Each phase lasts four months during the first study year and three months during the second. At the end of each study phase, ICUs are assigned to an intervention for a best practice not yet received according to a random schedule. The primary analysis will use patient-level process-of-care data to measure the intervention's effect on rates of adoption and adherence of each best practice in the targeted ICU clusters versus controls.

**Discussion:**

This study design evaluates a new system for knowledge translation and quality improvement across six common ICU problems. All participating ICUs receive quality improvement initiatives during every study phase, improving buy-in. This study design could be considered for other quality improvement interventions and in other care settings.

**Trial Registration:**

This trial is registered with  (ID #: NCT00332982)

## Background

The demand for intensive care is increasing because of an aging population and the introduction of new life-sustaining technologies[1]. This care is expensive and the necessary resources are limited [2-4]. Despite advances in critical care delivery, mortality remains high[5,6]. It is thus imperative that eligible patients receive interventions which improve outcomes or decrease intensive care unit (ICU) length of stay[7]. Delays between demonstration of effectiveness and the widespread use of such critical care evidence-based 'best practices'[8,9] constitute errors of omission and jeopardize patient outcomes[10,11]. These delays in implementation of clinical best practices may be more extreme in non-academic hospitals, with heavier individual clinician workloads and fewer personnel to engage in collaborative continuing educational activities. This general problem is compounded in the province of Ontario, Canada because ICUs are geographically widely separated and no formal quality improvement program exists[12]. Responding to these challenges, the Ministry of Health and Long-term Care sought proposals for development and evaluation of strategies to improve effectiveness of care in Ontario's health care system[13].

Changing clinical behaviour in the ICU can be challenging[14,15]. In the non-ICU setting, multifaceted interventions targeting different barriers to change are more likely to be effective than single interventions[16]. Promising strategies include educational outreach, audit and feedback, and reminders[17]. We hypothesize that a multifaceted knowledge translation approach among ICUs in a telemedicine network will increase the adoption of six evidence-based ICU clinical best practices that have been shown in high quality studies to improve patient care. The existing Ontario-wide videoconferencing telemedicine system allows all participants to communicate in real-time with each other and with the coordinating academic hospital. This study is registered at  (ID #: NCT00332982) [18].

## Methods and design

### Objective

Our objective is to evaluate the effectiveness of a novel knowledge translation program for increasing the proportion of patients who appropriately receive six evidence-based care practices. The effectiveness of this intervention will be considered at the level of individual patients and across clusters (ICUs) of patients.

### Participating ICUs

**Figure 1:**
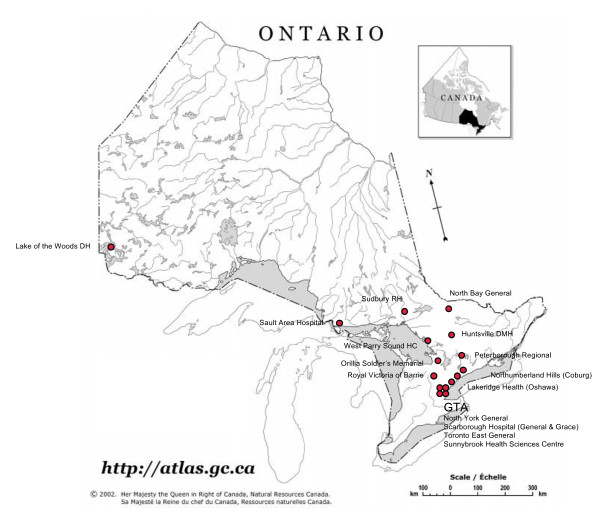
**Map showing geographic distribution of hospitals involved in the study network**. Map of Province of Ontario showing geographic locations of participating sites. Abbreviations: DH = District Hospital; RH = Regional Hospital; DMH = District Memorial Hospital; HC = Health Centre; GTA = Greater Toronto Area. Map reproduced with the permission of Natural Resources Canada, 2008, and Courtesy of the Atlas of Canada.

The study involves 15 Ontario community hospitals, with ICUs representing various geographic locations and ICU sizes (Figure 1). The network is centred at Sunnybrook Health Sciences Centre, where the medical-surgical-trauma ICU of this academic hospital will be used as a pilot site for the knowledge translation interventions and data collection approaches. Because this ICU already has a well-developed educational and quality improvement infrastructure, data collected from this academic ICU will not be considered in the primary analyses but will be included in secondary analyses. A central coordinating office will conduct the knowledge translation interventions, disseminate educational and promotional materials, arrange videoconferences, and analyze collected data. All participating ICUs are equipped with telemedicine videoconferencing equipment.

We conducted a baseline survey of the directors and nurse managers of participating ICUs to understand their organizational structure, quality improvement culture, and to obtain estimates of patient characteristics. Dedicated intensivists supervise the daily care of admitted patients ('closed' model) in seven (47%) ICUs, one (7%) ICU has intensivists available for consultation on admitted patients ('mixed' unit), and seven (47%) ICUs are staffed by generalists ('open' ICUs). Many ICUs conduct multidisciplinary rounds involving physicians (nine, 60%), nurses (11, 73%), respiratory therapists (nine, 60%), pharmacists (nine, 60%), and dieticians (seven, 47%). The estimated mean number of patients admitted annually to participating ICUs is 824 (range 307 to 1,700), and the median number of daily staffed and occupied beds is 10 (range four to 19). Mechanical ventilation is provided to an estimated 42% (range 10 to 65%) of patients.

### Selection of clinical best practices

We used the following criteria to select best practices: potential to improve clinical outcomes (based on existing evidence); applicable to most patients; feasible to implement and measure process of care indicators or patient outcomes; not already consistently applied. An expert advisory panel generated 15 candidate best practices believed to satisfy these criteria (Table 1). We then asked ICU directors of the participating sites (n = 15) to rate these at the time of our baseline survey. The following six best practices were chosen for this study because they received the highest ratings for relevance (four or five on a five-point Likert scale indicating 'very relevant' to 'extremely relevant') and mean estimated proportion of eligible patients: 1) prevention of venous thromboembolism (very to extremely relevant 87%; 72% of patients eligible); 2) prevention of ventilator-associated pneumonia (very to extremely relevant 80%; 50% of patients eligible); 3) prevention of catheter-related bloodstream infections (very to extremely relevant 93%; 76% of patients eligible); 4) daily use of spontaneous breathing trials for mechanically ventilated patients (very to extremely relevant 80%; 48% of patients eligible); 5) provision of early enteral nutrition (very to extremely relevant 87%; 70% of patients eligible); 6) and prevention of decubitus (pressure) ulcers (very to extremely relevant 93%; 68% of patients eligible).

**Table 1:** Candidate ICU clinical best practices considered for study

**Best Practice**
Prophylaxis against venous thromboembolism
Prevention of ventilator-associated pneumonia
Intensive insulin therapy to achieve tight glycemic control
Lung protective ventilation strategy
Daily interruption of sedation infusions
Restrictive transfusion strategy
Prophylaxis against gastric stress ulcers
Spontaneous breathing trials for mechanically-ventilated patients
Protocolized weaning from mechanical ventilation
Prevention of decubitus pressure ulcers
Provision of early enteral nutrition
Prevention of catheter-related blood stream infections
Pain assessment and management
Anxiety and delirium management
Improved end of life care

### Behaviour change strategies

We will use the following behaviour change strategies during the course of our study: educational outreach, audit and feedback, and reminders (Table 2)[17].

**Table 2:** Components of the multi-faceted knowledge translation intervention

**Intervention**	**Description**
Educational outreach	- Monthly videoconference with study coordinators to discuss progress and implementation strategies. - Educational sessions provided via videoconference by content experts for each best practice; available for later viewing on website - Development of a bibliography of evidence-based literature supporting each best practice - Summary of guidelines into easy to read bulletins - Support to local champions for presenting educational sessions.

Reminders	- Promotional items (posters, bulletins, pins, pens, stamps, pocket cards) - Pre-printed order sets - Checklists

Audit and feedback	- Daily audit of process of care indicators - Monthly reports of performance measures to each ICU during each phase - Each ICU performance compared anonymously to peer ICUs

### Educational outreach

For each best practice, a bibliography of relevant literature will be generated. Guidelines will be summarized in easy-to-read bulletins. A content expert will provide an interactive educational session using the videoconferencing network. These presentations will be made available on a website for later viewing. Each site will be encouraged to provide in-services and conduct their own educational activities.

### Audit and feedback

We will audit process of care indicators for each best practice (Table 3) on a daily basis and disseminate monthly feedback reports to participating ICUs. Each ICU will be able to determine the identity of their own hospital on these reports, but performance data from other hospitals will be presented as aggregate data. This will enable each ICU to perform anonymous inter-site comparisons to monitor their own progress throughout the project, and to provide feedback for educational and motivational purposes to their staff.

**Table 3:** Proposed best practice interventions and daily process of care indicators

**Best practice**	**Process of care indicator**	**Unit of measurement**
**Prevention of ventilator-associated pneumonia**	▪ head of bed elevation(≥ 30°) ▪ route of intubation	- Number of eligible patient-days with head elevation ≥ 30° - Number of eligible patient days associated with endotracheal intubation
		
**Prophylaxis against venous thromboembolism**	▪ administration of anticoagulant prophylaxis during first 48 hours ▪ use of antiembolic stockings if pharmacoprophylaxis contraindicated	- Number of eligible patients receiving appropriate anticoagulant prophylaxis
		
**Ventilator weaning strategy**	▪ spontaneous breathing trial or extubation within previous 24 hours	- Number of eligible patient-days on which spontaneous breathing trial (or extubation) was performed
		
**Prevention of catheter-related bloodstream infections**	▪ 7-point checklist for sterile insertion completed ▪ fulfilment of all 7 criteria listed on checklist ▪ anatomic site of catheter insertion	- Number of central venous catheters inserted using all 7 criteria on checklist - Number of central venous catheters inserted at the subclavian site
		
**Early enteral feeding**	▪ Initiation of enteral feeds within 48 hours of ICU admission	- Number of eligible patients receiving early enteral feeding within 48 hours of ICU admission - Number of eligible patients achieving 50% of their target caloric goal via the enteral route by 72 hours
		
**Pressure ulcer prevention**	▪ Completion of the Braden index at least daily ▪ Use of specialized mattress or bedding material to relieve pressure	- Number of patient days with Braden index completed - Number of patients receiving specialized mattress or bedding/all eligible patients

### Reminders

We will encourage the use of reminders to increase the use of each best practice. Examples of reminders include: promotional items (posters, bulletins, pins, pens, stamps, and pocket cards), pre-printed order sets, and checklists.

### Telemedicine

We will use the Ontario Telemedicine Network videoconferencing infrastructure, which allows for real-time and simultaneous interactive video discussions involving participants at multiple sites. We will use this network to coordinate study activities, provide interactive educational sessions from content experts, conduct monthly network meetings among ICUs, and host training sessions for data collectors and site educators.

### Study design

The study design will be a pragmatic cluster randomized trial with active control group[19]. Our intention is to enhance the application of evidence-based care practices by the whole ICU team, so randomization will occur at the level of clusters to minimise contamination [20-22]. In this study, these clusters will be the participating ICUs, because our intervention will target a group of healthcare providers and local infrastructure rather than individual clinicians or patients[23]. Our study is pragmatic, because it is being conducted specifically to evaluate the effectiveness of a quality improvement approach funded by the Ontario government[24,25]. Each ICU will receive active strategies to improve the use of a care practice, but will simultaneously function as a control for ICUs receiving an alternate care practice (active control).

### Randomization of participating ICUs and best practices

**Figure 2:**
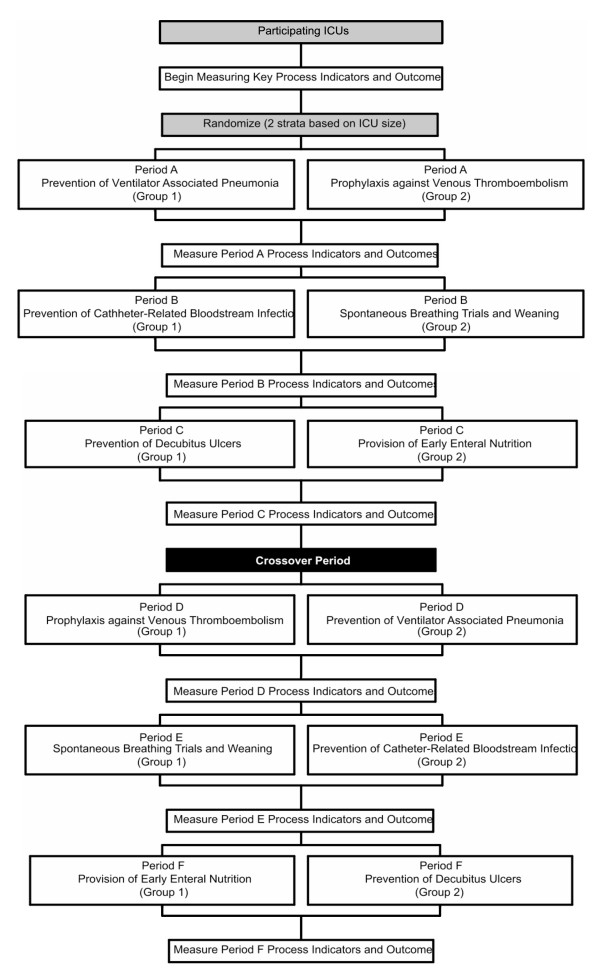
**Study flow**.

The 15 community ICUs will be randomly allocated into two groups (central computer-generated randomization with allocation concealment), with stratification by ICU size (≤ 10 versus > 10 staffed beds). The six best practices will be divided into the following three pairs to ensure that each does not impact on the same primary patient endpoint: 1) anticoagulation for prophylaxis against venous thromboembolism with semi-recumbent positioning to prevent ventilator-associated pneumonia; 2) sterile precautions for central venous catheter insertion to prevent catheter-associated bacteremia, with daily spontaneous breathing trials to decrease duration of mechanical ventilation; 3) early enteral nutrition with daily assessment of risk for developing decubitus (pressure) ulcers. These three pairs will be implemented in six phases according to a computer-generated random schedule, each four months long during the first year and, following the crossover point (Figure 2), three months long during the second year. The participating ICUs will be given their group assignments by the project coordinator at the start of each study phase. Although blinding within ICUs is not possible, they will be blinded to the other study arm's intervention.

### Data Collection

Collection of demographic information and pre-specified process of care indicators will be performed in each ICU by data collectors at times distinct from the usual multidisciplinary patient care rounds. These data collectors will all be trained by one study investigator (BH). A handheld electronic device (Palm Lifedrive™, Sunnyvale, CA, USA) will be used to collect data and will be synchronized in real time with the central database. Data collection will occur in all ICUs each day from Monday to Friday, but will be optional on weekends or holidays. Site visits and intermittent audits of data collection processes will be conducted by the central coordinating centre. All data entered into the handheld electronic devices will be automatically encrypted (128-bit) and wirelessly connected for real time uploading to the central encrypted database. All computers storing study-related information will require password entry and will be restricted to authorized members of the research team at all times.

The minimum demographic information collected from each patient is shown in Table 4. Each best practice will be associated with at least one process of care indicator (Table 3) and criteria for determining eligibility for the best practice. If a process of care indicator is recorded as being present, it will be assumed that the best practice has been delivered to that patient for that entire day. Once data collection for process of care has commenced for a specific best practice, these data will be collected in both study arms for the remainder of the study. However, the targeted campaign to improve any given best practice will only take place over three- to four-month periods.

**Table 4:** Minimum data set

**Variable**	**Data components**
Site number	Number
Unique identifier	Number
Date of birth	day/month/year
Sex	Female/male
Height	cm/inches
Hospital admission date	day/month/year
ICU admission date	day/month/year
ICU discharge date	day/month/year
Hospital discharge date	day/month/year
Patient classification	Medical/Surgical/Trauma
Death during hospitalization	yes/no; day/month/year

### Outcomes

The primary endpoint will be the difference in the rate of change in proportion of patients receiving each best practice in the actively targeted ICUs compared to the same practice in control ICUs during each four-month study phase. This will be described as the ratio of odds ratios for improvement over time for eligible patients receiving each best practice in the actively targeted ICUs compared to the same practice in control ICUs (odds ratio (actively targeted)/odds ratio (control)) and adjusted for clustering within centres. The unit of analysis for this endpoint will be the individual patient.

Secondary endpoints will include the rate of improvement over time in actively targeted ICUs and the rate of improvement over time in control ICUs; the proportion of eligible patients receiving each best practice during the final month of the study phase in active ICUs versus control ICUs; and the proportion of eligible patients receiving each best practice in actively targeted ICUs one year after the initial intervention. We will collect information on ICU length of stay and ICU mortality for descriptive purposes only. Limited clinical outcome measures will be measured for some best practices (for example, rate of catheter-related bloodstream infection). Health care worker satisfaction will be periodically measured using qualitative methodology.

### Data analysis

All data will be analyzed using SAS (version 9.1, Cary, NC). For descriptive statistics, we will report mean and standard deviation or median and inter-quartile range for continuous variables and proportions for dichotomous variables. We will use the student t-test or Mann Whitney U test, where appropriate, for comparisons of continuous variables and the Chi square or Fisher exact test for comparisons of proportions.

The odds ratio for receiving a particular best practice, identified using process of care indicators performed in eligible patients, will be calculated in both groups using generalized linear mixed methods (glmm) to account for the hierarchical nature (clustering within centres) of the data[26]. The primary dichotomous outcome, the rate of change in proportion of patients receiving each best practice, will be analyzed by testing for the effects of group (targeted intervention versus control), time (during four months of intervention), and the interaction between group and time (the ratio of the odds ratio of improving over time in the targeted group versus the odds ratio in the control group). For secondary analyses, a before-after comparison will be performed in all hospitals to calculate the odds ratio for receiving each best practice following the active intervention phase. For hospitals that have already been assigned to receive a particular best practice prior to the crossover period, we will be able to monitor for declining use of the best practice following the crossover point (reported as ratio of odds ratios for receiving each best practice, after versus before).

### Sample size

This study evaluates a planned quality improvement initiative involving a fixed number of ICUs over a defined funding period, so we performed a power rather than sample size calculation. Based on the results of our survey of ICU directors, we estimate that during a one-year period an average of 824 patients (range 307 to 1,700) will be admitted to each ICU. Since our study will involve 15 hospitals over two years, we expect that approximately 12,000 patients will be enrolled per study arm, and a total of 2,000 patients per four-month intervention phase. Assuming an average cluster size per phase of 250 patients and an intracluster (between centre) correlation coefficient (ρ) of 0.2 (variance inflation factor = 1 + (n-1)* ρ = 50; power = 80%; α = 0.05) [27], we should be able to detect a 20% increase when baseline adherence is 25%, a 30% increase when baseline adherence is 50%, or a 22% increase when baseline adherence is 75%.

### Ethics

This study has been approved by the Research Ethics Boards of all 16 participating hospitals, each of which has waived the requirement for obtaining individual patient consent.

## Discussion

The primary objective of this study is to determine whether a collaborative videoconferencing network to deliver a multifaceted knowledge translation intervention including education, reminders and audit and feedback can improve the care provided to critically ill patients across geographically separate ICUs. If successful, this study will provide a template for creating 'quality improvement clusters' of hospitals across regions, facilitating system-wide sharing of information and knowledge transfer, and delivering evidence-based clinical best practices to eligible patients.

To our knowledge, this is the first randomized controlled trial of a collaborative knowledge translation telemedicine network targeting adult ICU quality improvement. Others have used network approaches to improve care with variable results. Pronovost and colleagues conducted an uncontrolled before-after study of all ICUs in Michigan and showed a dramatic and sustained reduction in rates of catheter-related bloodstream infection[28]. The study was non-randomized and restricted its focus to only one care practice. Our study will incorporate some of the same knowledge-translation strategies for encouraging the sterile insertion of central venous catheters, but we will also target several other unrelated care practices. Martin and colleagues conducted a cluster-randomized trial of algorithms, including in-service education sessions, reminders, and academic detailing for improving the use of enteral nutrition in 14 ICUs[29]. The study was challenged by the crossover of two hospitals from the control to the intervention arm; these were then excluded from the primary analyses. Compared with the patients in the control hospitals (n = 214), the patients in the intervention hospitals (n = 248) received more days of enteral nutrition (6.7 versus 5.4 per 10 patient-days; p = 0.042) and had shorter mean stay in hospital (25 versus 35 days; p = 0.003), but had no significant difference in mortality or duration of ICU stay. We believe that videoconferencing communications may provide a more effective means of coordinating knowledge transfer interventions than the approach used by this study.

The organizational structure of an ICU can pose unique challenges to quality improvement because of the multidisciplinary approach to care, heterogeneous patient populations, and the focus on patients defined by geographical location in the hospital rather than by a particular disease [30-32]. Much has been written about the effectiveness of various knowledge translation strategies in the outpatient setting [17,33-37], but less is known about the ICU environment[38,39]. In a single-center qualitative study in two ICUs examining barriers to implementing semirecumbency to prevent ventilator-associated pneumonia, clinicians believed that a multifaceted approach involving education, guidelines, reminders, and audit and feedback could be important in changing clinician behaviour[40]. Horbar and colleagues conducted a cluster-randomized trial of 114 neonatal ICUs (which have similar organizational structure to adult ICUs) to evaluate a multifaceted strategy, including audit and feedback, evidence reviews, quality improvement training, and follow-up support to increase the delivery of surfactant therapy to eligible infants[41]. Their intervention was highly successful. Infants in intervention hospitals were more likely to receive surfactant in the delivery room and received the first dose of surfactant sooner after birth. Our study will adopt a similar approach, using multiple strategies for behaviour change, reasoning that these will be complementary and augment the effectiveness of individual knowledge translation components[16].

There is a need for new, more real-world knowledge translation studies to implement the flood of new evidence-based clinical practices[42]. However, a frequent shortcoming of many previous knowledge translation studies has been the use of non-randomized designs[43]. There is a need for pragmatic, cluster randomized trials to demonstrate the real-world relevance of these strategies at the level of individual ICUs[44]. Our innovative six-in-one trial of best practices implemented across a group of heterogeneous ICUs will enable evaluation of a collaborative, multi-faceted network intervention at the level of individual ICUs and at the level of the entire system.

The study design incorporates several unique features that merit attention. First, the cluster-randomized approach will enable inter- and intra-ICU comparisons of performance, and enable adjustment for unit-level factors that might affect utilization of best practices. Second, the active control arm ensures that all ICUs are engaged in quality improvement activities during each study phase (each ICU simultaneously functions as an intervention unit and a control unit), and avoids the perceptions of unfairness that would arise from randomizing individual ICUs to no quality improvement. Finally, the design allows for longitudinal before-after comparisons for units that are originally assigned to receive the control phase for a given best practice, and for assessment of decay in the use of a best practice for ICUs initially assigned to the intervention phase. We believe this unique study design should be appealing to policy makers and funding bodies interested in studying future system-level initiatives in the ICU and in other areas of healthcare.

## Competing interests

DS receives salary support for this research from the Ontario Ministry of Health and Long-term Care. BH and KD have been employed by the Ontario Ministry of Health and Long-term Care.

## Authors' contributions

DCS, KD, BH, RAF, NKJA, and MZ participated in the design of the study. DCS, KD, BH, RAF, NKJA, RP, and MZ planned the statistical analysis. All authors read and approved the final manuscript.
